# TREM2 expression in the brain and biological fluids in prion diseases

**DOI:** 10.1007/s00401-021-02296-1

**Published:** 2021-04-21

**Authors:** Daniela Diaz-Lucena, Niels Kruse, Katrin Thüne, Matthias Schmitz, Anna Villar-Piqué, Jose Eriton Gomes da Cunha, Peter Hermann, Óscar López-Pérez, Pol Andrés-Benito, Anna Ladogana, Miguel Calero, Enric Vidal, Joachim Riggert, Hailey Pineau, Valerie Sim, Henrik Zetterberg, Kaj Blennow, Jose Antonio del Río, Alba Marín-Moreno, Juan Carlos Espinosa, Juan María Torres, Raquel Sánchez-Valle, Brit Mollenhauer, Isidre Ferrer, Inga Zerr, Franc Llorens

**Affiliations:** 1 Network Center for Biomedical Research in Neurodegenerative Diseases (CIBERNED), L’Hospitalet de Llobregat, Spain; 2 Bellvitge Biomedical Research Institute (IDIBELL), L’Hospitalet de Llobregat, Spain; 3 University Medical Center Göttingen, Institute of Neuropathology, Göttingen, Germany; 4 Department of Neurology, University Medical Center Göttingen, Gern August University, Robert Koch Strasse 40, 37075 Göttingen, Germany; 5 German Center for Neurodegenerative Diseases (DZNE), Göttingen, Germany; 6 Keizo Asami Laboratory (LIKA), Universidade Federal de Pernambuco (UFPE), Recife, Brazil; 7 Department of Neurosciences, Istituto Superiore Di Sanità, Rome, Italy; 8 Alzheimer Disease Research Unit, CIEN Foundation, Chronic Disease Programme, Queen Sofia Foundation Alzheimer Center, Instituto de Salud Carlos III, Madrid, Spain; 9 Network Center for Biomedical Research in Neurodegenerative Diseases (CIBERNED), Madrid, Spain; 10 Centre de Recerca en Sanitat Animal, Campus Universitat Autònoma de Barcelona, Institut de Recerca I Tecnologia Agroalimentàries, Bellaterra, Spain; 11 Department of Transfusion Medicine, University Medical School, Göttingen, Germany; 12 Department of Medicine-Division of Neurology, Centre for Prions and Protein Folding Diseases, University of Alberta, Edmonton, Canada; 13 Department of Psychiatry and Neurochemistry, The Sahlgrenska Academy at the University of Gothenburg, Institute of Neuroscience and Physiology, Mölndal, Sweden; 14 Clinical Neurochemistry Laboratory, Sahlgrenska University Hospital, Mölndal, Sweden; 15 Department of Neurodegenerative Disease, UCL Institute of Neurology, London, UK; 16 UK Dementia Research Institute, London, UK; 17 Molecular and Cellular Neurobiotechnology, Scientific Park of Barcelona, Institute for Bioengineering of Catalonia (IBEC), The Barcelona Institute for Science and Technology (BIST), Barcelona, Spain; 18 Department of Cell Biology, Physiology and Immunology, Faculty of Biology, University of Barcelona, Barcelona, Spain; 19 Network Center for Biomedical Research in Neurodegenerative Diseases (CIBERNED), Barcelona, Spain; 20 University of Barcelona, Institute of Neuroscience, Barcelona, Spain; 21 Centro de Investigación en Sanidad Animal, CISA-INIA, Madrid, Spain; 22 Alzheimer’s Disease and Other Cognitive Disorders Unit, Neurology Department, Hospital Clinic de Barcelona, Institut D’Investigacions Biomediques August Pi I Sunyer (IDIBAPS), Barcelona, Spain; 23 Paracelsus-Elena Klinik, Kassel, Germany; 24 Department of Neurology, University Medical Centre Göttingen, Göttingen, Germany; 25 Department of Pathology and Experimental Therapeutics, Hospitalet de Llobregat, University of Barcelona, Feixa Llarga S/N, 08907 Barcelona, Spain

**Keywords:** TREM2, Prion diseases, Creutzfeldt-Jakob disease, Microglia, Cerebrospinal fluid, Plasma

## Abstract

Triggering receptor expressed on myeloid cells 2 (TREM2) is an innate immune cell surface receptor that regulates microglial function and is involved in the pathophysiology of several neurodegenerative diseases. Its soluble form (sTREM2) results from shedding of the TREM2 ectodomain. The role of TREM2 in prion diseases, a group of rapidly progressive dementias remains to be elucidated. In the present study, we analysed the expression of TREM2 and its main sheddase ADAM10 in the brain of sporadic Creutzfeldt-Jakob disease (sCJD) patients and evaluated the role of CSF and plasma sTREM2 as a potential diagnostic marker of prion disease. Our data indicate that, compared to controls, TREM2 is increased in sCJD patient brains at the mRNA and protein levels in a regional and subtype dependent fashion, and expressed in a subpopulation of microglia. In contrast, ADAM10 is increased at the protein, but not the mRNA level, with a restricted neuronal expression. Elevated CSF sTREM2 is found in sCJD, genetic CJD with mutations E200K and V210I in the prion protein gene (*PRNP*), and iatrogenic CJD, as compared to healthy controls (HC) (AUC = 0.78–0.90) and neurological controls (AUC = 0.73–0.85), while CSF sTREM2 is unchanged in fatal familial insomnia. sTREM2 in the CSF of cases with Alzheimer’s disease, and multiple sclerosis was not significantly altered in our series. CSF sTREM2 concentrations in sCJD are *PRNP* codon 129 and subtype-related, correlate with CSF 14-3-3 positivity, total-tau and YKL-40, and increase with disease progression. In plasma, sTREM2 is increased in sCJD compared with HC (AUC = 0.80), displaying positive correlations with plasma total-tau, neurofilament light, and YKL-40. We conclude that comparative study of TREM2 in brain and biological fluids of prion diseases reveals TREM2 to be altered in human prion diseases with a potential value in target engagement, patient stratification, and disease monitoring.

**Supplementary Information:**

The online version contains supplementary material available at 10.1007/s00401-021-02296-1.

## Introduction

Prion diseases (or transmissible spongiform encephalopathies) are a rare group of invariably fatal neurodegenerative dementias characterized by a rapid progression affecting humans and animals. The causative agent of prion diseases is the pathological form of the cellular prion protein (PrP^c^), PrP^Sc^, which nucleates and self-propagates in the central nervous system (CNS) [3]. However, the mechanisms by which PrP^Sc^ causes neurodegeneration are not well-understood.

Human prion diseases can be classified according to their aetiology as sporadic Creutzfeldt-Jakob disease (sCJD); genetic Creutzfeldt-Jakob disease (gCJD), Gerstmann-Sträussler-Scheinker syndrome (GSS), and fatal familial insomnia (FFI); and as iatrogenic Creutzfeldt-Jakob disease (iCJD), variant Creutzfeldt-Jakob disease (vCJD), and Kuru. sCJD is the most prevalent human prion disease and its typical clinical phenotype includes rapidly progressive dementia, behavioural abnormalities, ataxia, extrapyramidal features, and myoclonus. Eventually, sCJD patients fall into a state of akinetic mutism before death [18, 54]. Neuropathologically, sCJD is characterized by neuronal loss, spongiform change, reactive astrocytosis, and microgliosis. These changes are accompanied by the accumulation of a protease-resistant form of host-derived prion protein (PrP^res^) [17].

A clinical diagnosis of probable sCJD is achieved by the combination of clinical symptoms and signs, neuroimaging, and cerebrospinal fluid tests (CSF) [23, 26]. The most relevant CSF biomarkers are surrogate neuronal damage markers 14-3-3, total-tau (t-tau), and α-synuclein (a-syn), while the real-time quaking induced conversion (RT-QuIC) assay is a prion-specific assay allowing identification of the pathological prion protein in the CSF [70]. Recently, some blood biomarkers have been shown to be suitable in discriminating sCJD cases but their accuracy in the differential diagnosis of sCJD has not been determined [30, 66, 72]. Therefore, they are not yet included in clinical practice. Importantly, the use of surrogate CSF biomarkers is not limited to diagnostic purposes; rather, a potential use as prognostic, predictive, monitoring, and response biomarkers has been proposed [35, 46, 65, 71]. Therefore, the characterization of the best putative application for each biomarker as well as the identification of biomarkers targeting the complete pathological signatures of prion disease will greatly enhance the rational development and interpretation of future therapeutic interventions. In this regard, the observation that exacerbated inflammatory profiles is an early pathogenic event in prion diseases [2, 34] has led to the search for surrogate biomarkers of neuroinflammation for early diagnosis. In this context, glial-specific proteins such as the S100 calcium-binding protein B (S100B), chitinase-3-like protein 1 (CHI3L1/YKL-40), chitotriosidase 1 (CHIT1), and the glial fibrillary acidic protein (GFAP) have been reported to be increased in the CSF of sCJD [2, 25, 37, 58].

In recent years, strong evidence of a link between TREM2 function in the innate immune system and the pathogenesis of neurodegenerative diseases has been documented. Although rare in the population, certain TREM2 variants are associated with increased risk of developing neurodegenerative diseases such as Alzheimer’s disease (AD) [21, 26], frontotemporal dementia [20, 56], Parkinson’s disease [56], and amyotrophic lateral sclerosis [7], presumably as a consequence of TREM2 loss of function [12]. In this regard, TREM2 mutations led to reduced phagocytic function [64], while injection of sTREM2 in an AD mouse model enhanced microglial proliferation, migration, clustering in the vicinity of amyloid plaques, and the uptake and degradation of β-amyloid [79]. In AD patients, increased TREM2 levels have been reported in the CSF [22, 24, 41, 42, 55], together with positive immunoreactivity in the microglia associated with β-amyloid plaques and in neuritic pathology-enriched areas [41]. It is currently accepted that TREM2 is expressed predominantly in cells of myeloid lineage, in line with its role in brain innate immune function [15, 28, 61]. No effect of genetic variation in *TREM2* in the risk of CJD has been identified [63]. Yet pioneering studies have shown increased microglial *TREM2* mRNA expression in scrapie-infected mice and prion-inoculated *TREM2* knockout mice, with attenuated microglia response compared to controls but no alterations in prion pathogenesis in terms of disease onset, disease duration, and presence of pathological PrP [80]. TREM2 expression in brain and biological fluids of human prion diseases and its potential role as a candidate biomarker of prion pathogenesis have not been explored.

## Material and methods

### Antibodies and reagents

Human TREM2 goat polyclonal antibody (RD Systems, AF1828) was used diluted 1:20 for immunohistochemistry and at a dilution of 1:200 for western blotting. The antibody anti-TREM2 Abcam [EPR20243] ab209814 diluted 1:100 was used for immunohistochemistry for comparative purposes. Human ADAM10 antibody (Abcam, ab1997) was used at a dilution of 1:75 for immunohistochemistry and 1:500 for western blotting. Monoclonal anti-β-actin antibody (Sigma-Aldrich, A5316) was used at 1:30,000 as a protein-loading control in western blotting. For double-labelling immunofluorescence and confocal microscopy visualization, TREM2 was combined with the microglial marker IBA1 diluted 1:1000 (Wako, 019-19,741).

### Brain samples

Brain tissue was obtained from the Institute of Neuropathology HUB-ICO-IDIBELL Biobank and the Hospital Clinic de Barcelona-IDIBAPS Neurological Tissue Bank following the Spanish legislation guidelines on this matter (Real Decreto de Biobancos 1716/2011) and approval of the local ethics committees. Processing of brain tissue has been detailed elsewhere [16]. The post-mortem interval between death and tissue processing was between 1 h 30 min and 24 h. One hemisphere was immediately cut in coronal sections, 1-cm thick, and selected areas of the encephalon were rapidly dissected, frozen on metal plates over dry ice, placed in individual air-tight plastic bags, numbered with water-resistant ink, and stored at − 80 °C until use for biochemical studies. The other hemisphere was fixed by immersion in 4% buffered formalin for 3 weeks for morphologic study. Neuropathological examination was performed in every case on formalin-fixed, paraffin-embedded samples. De-waxed paraffin Sects. 4 microns thick were stained with haematoxylin and eosin, periodic acid Schiff, Congo red, and Klüver-Barrera, or processed for immunohistochemistry using anti-β-amyloid, p-tau (clone AT8), α-synuclein, αB-crystallin, TDP-43, P-TDP-43, ubiquitin, p62, glial fibrillary acidic protein, CD68, and IBA1, as previously reported [16]. Neuropathological diagnosis of Alzheimer’s disease (AD) was categorized following Braak stages of neurofibrillary tangle pathology adapted to paraffin sections [5, 6]. Cases with Frontotemporal lobar degeneration with TDP-43 pathology (FTLD-TDP) were diagnosed following established neuropathological criteria [43]. Post-mortem neuropathological examination confirmed the diagnosis of sCJD cases according to established criteria [49, 50]. The analysis of the codon129 genotype of PrP gene (*PRNP*) (methionine or valine) was performed after isolation of genomic DNA from blood samples according to standard methods. sCJD type 1 or type 2 classification was performed with western blotting. Cases with associated pathologies such as vascular diseases, infections of the nervous system, neoplastic diseases affecting the nervous system, systemic and central immune diseases, metabolic syndrome, hypoxia, and prolonged axonal states such as those occurring in intensive care units were excluded from the present study.

Since a percentage of CJD cases have concomitant NFT pathology at stages 0-III of Braak, with occasional amyloid plaques (stage A of Braak), control cases in the present series are not controls without NFT pathology but rather age-matched controls with NFT pathology at stages 0-III without neurological symptoms. Mild small blood vessel disease and occasional status cribosus occurred in the ‘control’ and ‘pathological’ groups.

Molecular studies were carried out in frontal cortex area 8 (FC) and the cerebellum (CB). The CJD group was composed of controls (*n* = 15 FC, *n* = 12 CB), sCJD (*n* = 11 FC, *n* = 12 CB), and sCJD VV2 (*n* = 11 FC, *n* = 10 CB). The sFTLD group consisted of samples from the FC of controls (*n* = 17) and sFTLD-TDP (*n* = 16). Details of the brain samples used for q-PCR are summarized in Supplementary Table 1. Samples for western blotting were obtained from 10 controls, 8 sCJD MM1, and 8 sCJD VV2 cases.

Triggering receptor expressed on myeloid cells 2 immunohistochemistry was performed on de-waxed paraffin sections of controls (*n* = 10; 7 M, 3 F; mean age ± SD: 57.9 ± 12.5), CJD MM1 (*n* = 7; 6 M, 1 not available; mean age ± SD: 63.8 ± 13.9), AD V-VI (*n* = 3; 3 F; mean age ± SD: 77.3 ± 4.1), sFTLD-TDP (*n* = 3; 3 M; mean age ± SD: 72 ± 7.1), DLB (*n* = 3; 2F,1 M; mean age ± SD: 78.3 ± 4.9), ALS (*n* = 4; 3 F, 1 M; mean age ± SD: 62 ± 12.5), white matter with plaques of relapsing remitting cases of multiple sclerosis (MS, *n* = 1 M, 52 years), and vascular dementia with subacute areas of infarction (DM, *n* = 3 M; 72) (see Supplementary table 3).

### Cerebrospinal fluid and plasma samples

Cerebrospinal fluid (CSF) samples (Table 1) from healthy control individuals (HC, *n* = 48), patients with non-primarily neurodegenerative neurological diseases (ND, *n* = 64), AD (*n* = 35), and sCJD (*n* = 139) were collected at the Clinical Dementia Centre and the National Reference Centre for CJD Surveillance at the University Medical Centre of Göttingen (Germany) and at the Alzheimer’s Disease and Other Cognitive Disorders Unit, Hospital Clínic de Barcelona, Barcelona (Spain). Patients with sCJD, classified as probable (*n* = 47) or definite (*n* = 92) cases according to diagnostic consensus criteria [76], genetic CJD (*n* = 91), iatrogenic CJD (*n* = 10), and fatal familial insomnia (FFI, *n* = 26) were collected in the following CJD reference centres: (1) Clinical Dementia Centre and the National Reference Centre for CJD Surveillance at the University Medical Centre, Göttingen, Germany, (2) Alzheimer’s Disease and Other Cognitive Disorders Unit, Hospital Clínic, Barcelona, Spain, (3) National Centre of Microbiology-Carlos III Institute of Health, Madrid, Spain, and (4) Istituto Superiore di Sanità, Rome, Italy. The diagnosis of genetic prion diseases was carried out according to surveillance criteria after prion protein gene (*PRNP*) analysis [31] and following World Health Organization (WHO) criteria [73]. Iatrogenic CJD was diagnosed according to established WHO criteria [73]. Nine iatrogenic cases were associated with dura mater grafts and one with corneal transplantation. AD cases were diagnosed according to the National Institute on Aging—Alzheimer’s Association workgroups (NIA-AA) criteria [45].

**Table 1:** Biomarker characteristics of the cases used in the CSF cohort of study. Diagnostic groups included controls (HC, ND), prion diseases (sCJD, gCJD-E200K, gCJD-V210I, FFI and iCJD), and AD cases

	*n*	Sex (f/m)	Age	sTREM2 (pg/mL)	95% CI	tau (pg/mL)	14-3-3	*p* value	
			Mean + SD	Mean + SD		Mean + SD	Pos/neg	sTREM2 vs. sex	sTREM2 vs. age
HC	48	26/22	64 ± 15	1898 ± 842	1653–2143	246 ± 132	0/48	0.26	0.20
ND	64	39/25	66 ± 9	2321 ± 1125	2040–2602	441 ± 410	13/49	0.87	0.61
sCJD	139	73/66	66 ± 11	6975 ± 8523	5544–8403	7337 ± 7658*	118/21	0.83	0.26
gCJD—E200K	57	35/22	60 ± 10	6341 ± 8128	4184–8498	6548 ± 5688	49/8	0.13	0.91
gCJD—V210I	34	18/16	66 ± 10	8231 ± 10,257	4652–11,810	10,548 ± 9985	27/7	0.18	0.63
FFI—D178N-M	26	9/17	50 ± 12	2871 ± 2704	1779–3963	993 ± 1715	4/22	0.22	0.19
iCJD	10	5/5	50 ± 11	4709 ± 2712	3025–6036	12,171 ± 10,053	8/2	0.42	0.44
AD	35	22/13	67 ± 10	2896 ± 1603	2345–3447	633 ± 401	NA	0.41	0.25

Number of cases (*n*), age [mean value ± standard deviation (SD)], sex distribution (*f* female, *m* male), CSF sTREM2 [mean value ± SD, and 95% confidence interval (95% CI)] and *t*-tau (mean value ± SD) concentrations as well as 14-3-3 positivity (pos/positive, neg/negative) are indicated. Inconclusive 14-3-3 tests were considered as negative. Significant associations (*p* value) between sTREM2 with sex and age for each diagnostic group are reported. *For 13 cases, t-tau data were not available. (*NA* not available)

In addition, CSF was obtained from patients with relapsing/remitting multiple sclerosis [69] at the Multiple Sclerosis Unit, Service of Neurology, Bellvitge University Hospital (*n* = 16), and the Clinical Dementia Centre and the National Reference Centre for CJD Surveillance at the University Medical Centre of Göttingen (Germany) (*n* = 13 (Supplementary table 4).

Cerebrospinal fluid tests in healthy controls (HC) was obtained from patients for knee surgical procedures under spinal anaesthesia.

The ND group was composed of cases diagnosed with neurological diseases not associated with neurodegenerative pathology and included the following diagnostic groups: psychosis, paranoid psychosis, bipolar disorder, schizophrenia, ischemic stroke, multiple infarct, epilepsy, meningitis, alcohol abuse, vertigo, acute or chronic headache, pain syndromes, acute hypoxia, polyneuropathy, cerebral lymphoma, astrocytoma, and paraneoplasic syndromes. ND cases were diagnosed according to acknowledged standard neurologic clinical and para-clinical findings based on the International Classification of Diseases 10th Revision definitions.

Blood was collected in Plasma-EDTA tubes at the Department of Transfusion Medicine (healthy controls, HC; *n* = 97 cases) and at the Department of Neurology (sCJD cases, *n* = 86 cases) of the University Medical Centre Göttingen (Germany) under standardized pre-analytical conditions. The healthy control (HC) group was composed of healthy blood donors without any relevant clinical findings (Table 2).

**Table 2:** Demographic and biomarker characteristics of the cases used in the plasma cohort study

	*n*	Sex (f/m)	Age	sTREM2 pg/mL)	95% CI	*p* values	
			Mean + SD	Mean + SD		sTrem2 vs. Sex	sTrem2 vs. age
HC	97	23/74	64 ± 5	4409 ± 3961	3611–5208	0.58	0.12
sCJD	86	52/34	65 ± 8	8981 ± 8932	7066–10,896	0.94	0.11

Diagnostic groups included HC and sCJD. Number of cases (*n*), age: mean value ± standard deviation (SD), sex distribution (*f* female, *m* male), and plasma sTREM2 concentrations: mean value ± SD and 95% confidence interval (95% CI) are indicated. Significant associations (*p* value) between sTREM2 with sex and age for each diagnostic group are indicated

For the analysis of CSF sTREM2 concentrations at different disease stages in sCJD, we divided the time from lumbar puncture (LP) or blood uptake to disease onset in each patient by the total duration of the disease. Then, samples were grouped into three categories according to whether they underwent LP/blood uptake in the first (time of LP to disease onset/total duration of the disease < 0.33), second (0.33–0.66), or third (> 0.66) stage of the disease, as previously reported [59].

### CSF and plasma tests

Soluble TREM2 (sTREM2) concentrations in the CSF and plasma were quantified with a previously described immunoassay [29] using the electrochemiluminescence-based MesoScale Discovery platform. Inter- and intra-assay coefficients of variation in our study were below 12% and 10%, respectively. CSF from MS cases was assessed in two series. In the first, the method was the previously described immunoassay [29], while for the second series (Bellvitge) assessment was made using the Human TREM2 ELISA Kit (Abcam, ab224881). CSF total-tau (t-tau) was quantified using a commercially available colorimetric enzyme-linked immunosorbent assay (INNOTEST hTAU-Ag from Fujirebio). CSF was analysed for the presence of 14 to 3-3 protein using western blotting [75]. CSF and plasma YKL-40 were quantified using the MicroVue YKL-40 ELISA kit from Quidel. Plasma neurofilament light (NfL) and t-tau concentrations were measured using commercially available assays on the Single molecule array (SIMOA) HD-1 instrument (Quanterix). The analysts were masked to clinical data.

### Animal experimentation

We conducted animal experiments in accordance with the Code for Methods and Welfare Considerations in Behavioural Research with Animals (Directive 2010/63/EU) and made every effort to minimize suffering. Experiments developed in the Centro de Investigación en Sanidad Animal–Instituto Nacional de Investigación y Tecnología Agraria y Alimentaria (Madrid, Spain) were evaluated by the Committee on the Ethics of Animal Experiments of the Instituto Nacional de Investigación y Tecnología Agraria y Alimentaria and approved by the General Directorate of the Madrid Community Government (permit no. PROEX 094/18). The tg340 and tg361 mouse lines expressing about four-fold level of human PrPM129 and PrPV129 on a mouse PrP null background were used as mouse models of sCJD MM1 and sCJD VV2 subtypes. Individually identified 6-to-10-week-old mice were anaesthetized and inoculated with 2 mg of a 10% (w/v) brain homogenate of sCJD MM1 (in the tg340 mouse line) and sCJD VV2 (in the tg361) in the right parietal lobe using a 25-gauge disposable hypodermic needle. Control animals for each genotype were inoculated with control brain homogenates. Mice were observed daily and their neurological status was assessed weekly. At 80, 120, or 180 days post-inoculation (dpi) animals were euthanized, necropsy was performed, and the brain was removed. Four to eight animals per time point and condition were used for RT-qPCR assays.

### Western blotting

Human tissues were lysed in RIPA lysis buffer RIPA (NaCl 1 M, Nonidet P-40 1%, sodium deoxycholate 0.5%,SDS 0.1%, Tris 50 mM, pH 7.4) supplemented with protease, and phosphatase inhibitors (Roche, GE). After centrifugation at 14,000*g* for 20 min at 4 °C, supernatants were quantified for protein concentration (BCA, Pierce), mixed with SDS-PAGE sample buffer, boiled, and subjected to 4–12% BisTris Gels (NuPAGE^™^ 4 to 12%, Bis–Tris, 1.5 mm, Mini Protein Gel, 15-well, ThermoFisher NP0336BOX). Gels were transferred onto PVDF membranes and processed for specific immunodetection. Membranes were incubated overnight at 4 °C with primary antibodies and with appropriate horseradish peroxidase-conjugated secondary antibody (anti-mouse/anti-rabbit HRP-IgG) diluted at 1:2000 (Dako) for 1 h at room temperature. Chemiluminescence immunodetection was performed using the ECL-Amersham (GE Healthcare) kit. Densitometry was carried out with the ImageJ software and values were normalized using β-actin levels.

### Real-time polymerase chain reaction (qPCR)

RNA from different human and mouse brain regions was purified using RNeasy Plus Mini Kit (Qiagen, 74,136), which eliminates genomic DNA contamination. RNA integrity was assessed with the RNA Integrity Number (RIN value) determined with the Agilent 2100 Bioanalyzer (Agilent) (RIN values between 4 and 9.2). Reverse transcription of the RNA samples was carried out using the High-Capacity cDNA Reverse Transcription kit (Applied Biosystems) following the supplier’s instructions. Quantitative RT-PCR assays were performed in duplicate on cDNA samples in 384-well optical plates with ABI Prism 7900 Sequence Detection System (Applied Biosystems, Life Technologies). The reactions were carried out using 20xTaqMan Gene Expression Assays and 2xTaqMan Universal PCR Master Mix (Applied Biosystems) with the following probes: CD68 Hs00154355_m1, TREM2 human Hs00219132_m1, TREM2 mouse Mm04209424_g1, and ADAM10 human Hs00153853_m1. The reactions were conducted using the following parameters: 50 °C for 2 min, 95 °C for 10 min, and 40 cycles with 95 °C for 15 s and 60 °C for 1 min. The fold change was determined by the ΔΔCt method, in which each target gene is normalized to its endogenous control to obtain the ΔCt. Afterwards, each ΔΔCt is determined by subtracting the mean-ΔCt of the control group samples to the ΔCt of each sample. Finally, these ΔΔCt values are used as the negative exponent with base 2 to obtain the fold change per sample. Mean fold change values were analysed with appropriate statistical test indicated in each figure using GraphPad Prism 6.01. Gene expression levels were normalized to the housekeeping genes Glucuronidase beta (GUSB) Hs00939627_m1 NM_000181.3 or glyceraldehyde 3-phosphate dehydrogenase (GAPDH) Hs03929097_g1 NM_001289745.1 for human and, GAPDH Mm99999915_g1 NM_001289726.1 for mouse (Applied Biosystems).

### Immunohistochemistry

De-waxed sections, 4 microns thick, were processed for immunohistochemistry. The sections were boiled in citrate buffer to retrieve antigenicity, followed by endogenous peroxidase blockage (Dako Real peroxidase blocking solution S2023). All sections were incubated overnight at 4 °C with primary antibodies diluted in Dako Real Antibody Diluent (Dako S2022).

Primary antibody anti-human TREM2 (RD Systems AF1828) diluted 1:20, anti-human TREM2 (Abcam, ab209814) diluted 1:100, and anti-human ADAM10 (Abcam, 1997) diluted 1:150 were used. Detection was achieved by incubation with R.T.U. Biotinylated Universal Antibody (Abcam ab64257) for 10 min at room temperature, followed by R.T.U. HRP-Streptavidin (Abcam ab64269). The peroxidase reaction was visualized with diaminobenzidine and H_2_O_2_. Control of the immunostaining included omission of the primary antibody.

### Immunofluorescence

De-waxed sections, 4 microns thick, were boiled in citrate buffer to enhance antigenicity, and afterwards were stained with a saturated solution of Sudan black B (Merck, DE) to block auto-fluorescence of lipofuscin granules present in cell bodies. Then they were rinsed in 70% ethanol and washed in distilled water. All sections were blocked at room temperature with 10% foetal bovine serum diluted in PBS, and incubated at 4ºC overnight with combinations of primary antibodies. After washing, the sections were incubated with Alexa-488 and Alexa-555 (Molecular Probes, Eugene, Oregon, USA) fluorescence secondary antibodies. Nuclei were stained with DRAQ5^™^ (dilution 1:2,000, BioStatus, Loughborough, UK). After washing, the sections were mounted in Immuno-Fluore mounting medium (ICN Biomedicals, Irvine, CA, USA), sealed, and dried overnight. Sections were examined with a Leica TCS-SL confocal microscopy.

### Statistical analysis

For brain tissue data, in two group comparisons, non-parametric Mann–Whitney-*U* tests were used. For comparisons between multiple groups, Kruskal–Wallis tests followed by Dunn’s post-hoc test or one-way ANOVA followed by Tukey post-hoc test were applied after testing for normality. Biomarker data were log-transformed, and linear models were built controlling for the effect of age and sex. To assess the diagnostic accuracy of sTREM2, receiver operating characteristic (ROC) curve analyses were carried out and areas under the curve (AUC) with confidence intervals (95% CI) were calculated. Spearman rank correlation coefficients were used to assess associations between continuous biomarker levels. Statistical comparison between ROC curves was made with the pROC R package [57]. Longitudinal analysis of sTREM2 along disease stages was conducted with nlme R package [53] using a multilevel linear model that assumed a fixed slope for disease stage.

## Results

### TREM2 and ADAM10 expression in the brain of sCJD patients

**Fig. 1:**
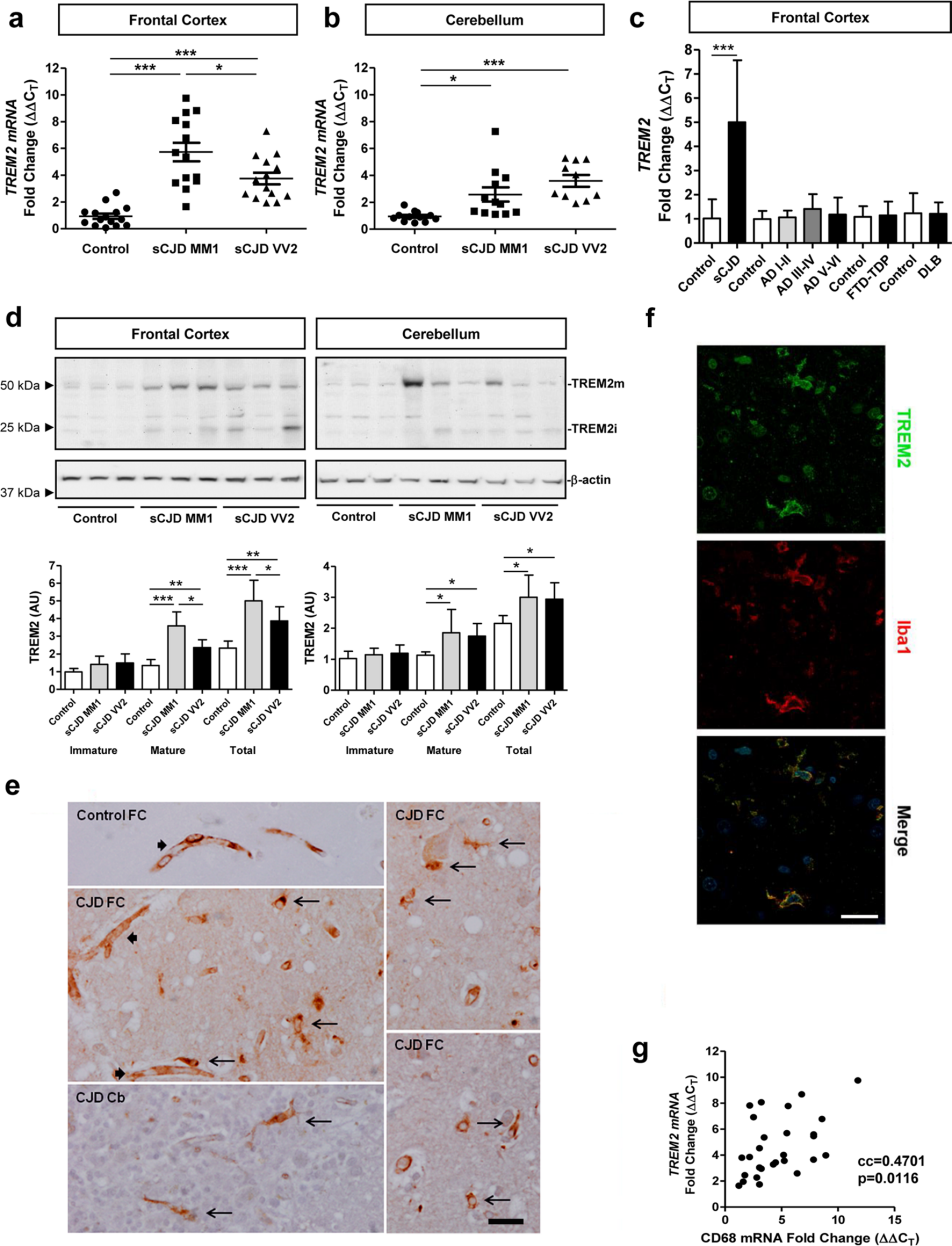
TREM2 expression in sCJD brain tissue**.** RT-qPCR analysis of *TREM2* in: **a** the frontal cortex (FC) and **b** cerebellum (CB) of control, sCJD MM1, and sCJD VV2 cases. *GAPDH* was used for normalization. FC (controls *n* = 15, sCJD MM1; *n* = 11, sCJD VV2; *n* = 11), CB (controls *n* = 12, sCJD MM1; *n* = 12, sCJD VV2; *n* = 10). **c** RT-qPCR analysis of *TREM2* in the FC of sCJD (MM1 and VV2), and FTD-TDP. For each neurodegenerative dementia, data were compared to age and sex-matched controls. CJD (controls *n* = 14, sCJD (MM1 and VV2); *n* = 22), FTD-TDP-43 (controls *n* = 17, FTD-TDP-43; *n* = 16). **d** Representative western blot analysis of TREM2 in the FC and CB of control, sCJD MM1, and sCJD VV2 cases. TREM2 immature (TREM2i) and mature (TREM2m) forms are indicated. For normalization β-actin was used. Graphical representation of western blot data acquired from the analysis of FC (controls *n* = 8, sCJD MM1; *n* = 8, sCJD VV2; *n* = 10) and CB (controls; *n* = 8, sCJD MM1; *n* = 8, sCJD VV2; *n* = 8) is shown; *p < 0.05, **p < 0.01, ***p < 0.001. **e** TREM-2 immunohistochemistry in frontal cortex and cerebellum in control cases, and then in frontal cortex in CJD MM1. In control and CJD cases, TREM2 immunoreactivity is localized in the blood vessels (thick arrows); in CJD, TREM2 immunoreactivity is also observed in glial cells with the morphology of microglia (long thin arrows); scale bar = 30 µm. **f** TREM2 double-labelling immunofluorescence and confocal microscopy in FC of sCJD showing TREM2 localization in a subset of microglial cells identified with the antibody Iba1 (white arrows). Nuclei are stained with DRAQ5^™^ (blue); scale bar = 20 µm. **g** Correlation analysis between *TREM2* and *CD68* mRNA in the FC of sCJD cases (*n* = 28). cc: correlation coefficient

Expression of *TREM2* was analysed in the frontal cortex (FC) and cerebellum (CB) of sCJD MM1 and VV2 cases. With RT-qPCR analysis, *TREM2* mRNA was significantly increased in the FC of sCJD MM1 (*n* = 14, *p* < 0.001, fold change 6.1) and VV2 (*n* = 14, *p* < 0.001, fold change 3.5) cases compared to controls (*n* = 15). *TREM2* mRNA expression was higher in sCJD cases possessing the *PRNP* gene codon 129 MM1 as compared to VV2 genotype (*p* < 0.05) (Fig. 1a). In the CB, *TREM2* mRNA was significantly increased in sCJD VV2 (*n* = 10, *p* < 0.001) and MM1 (*n* = 12, *p* < 0.05) compared to controls (*n* = 12) (Fig. 1b). *TREM2* levels were normalized to *GAPDH*, and similar results were obtained for *GUSB* normalization (data not shown). Increased *TREM2* mRNA expression in the FC in sCJD was significant (*n* = 28, *p* < 0.001) when compared with FTLD-TDP (*n* = 16) (Fig. 1c).

Western blot analysis revealed significantly increased protein levels of mature and total TREM2 (mature plus immature forms) in the FC of sCJD cases compared to controls (*p* < 0.001, control vs. sCJD MM1 and *p* < 0.01, control vs. sCJD VV2 for total TREM2) (Fig. 1d). The expression of mature and total TREM2 was significantly higher in sCJD MM1 cases compared to sCJD VV2 (*p* < 0.05). In the CB, mature and total TREM2 was significantly higher in sCJD cases compared to controls (*p* < 0.05 in all comparisons) (Fig. 1d). In both brain regions, 10 controls, 8 sCJD MM1, and 8 sCJD VV2 cases were used.

In immunohistochemistry study of control brains using the TREM2 polyclonal RD Systems, AF1828H antibody disclosed immunostaining in the blood vessel walls, with no immunostaining of neurons or glial cells (data not shown). Using the same antibody, TREM2 immunoreactivity in sCJD MM1 brains was observed in the blood vessel walls and in microglia; neurons were not immunostained with this antibody (Fig. 1e). De-waxed paraffin sections of AD, DLB, MS, sALS, and VD cases showed TREM2 immunoreactivity in the blood vessels and microglia surrounding β-amyloid plaques in AD, lipid-laden macrophages in subacute MS plaques, macrophages and a few mononuclear cells in subacute infarcts, and a few microglia in the spinal cord in sALS. Neurons were not stained with this antibody (data not shown). To validate immunohistochemical data, double-labelling immunofluorescence against TREM2 and Iba-1 in the FC of sCJD MM1 cases disclosed TREM2 localization in a subpopulation of microglial cells as revealed with the IBA1 antibody (Fig. 1f). Additionally, *TREM2* mRNA in FC of sCJD cases correlated positively with the macrophage marker *CD68* (*n* = 28, cc = 0.4701, *p* = 0.0116) (Fig. 1g).

In contrast, immunohistochemistry with the anti-TREM2 Abcam antibody showed neuronal immunoreactivity in control and diseased cases, in addition to immunostaining of the blood vessel walls and rare microglia (data not shown). We tried to reproduce these observations using another batch but the TREM2 Abcam antibody is a discontinued product and no longer available.

**Fig. 2:**
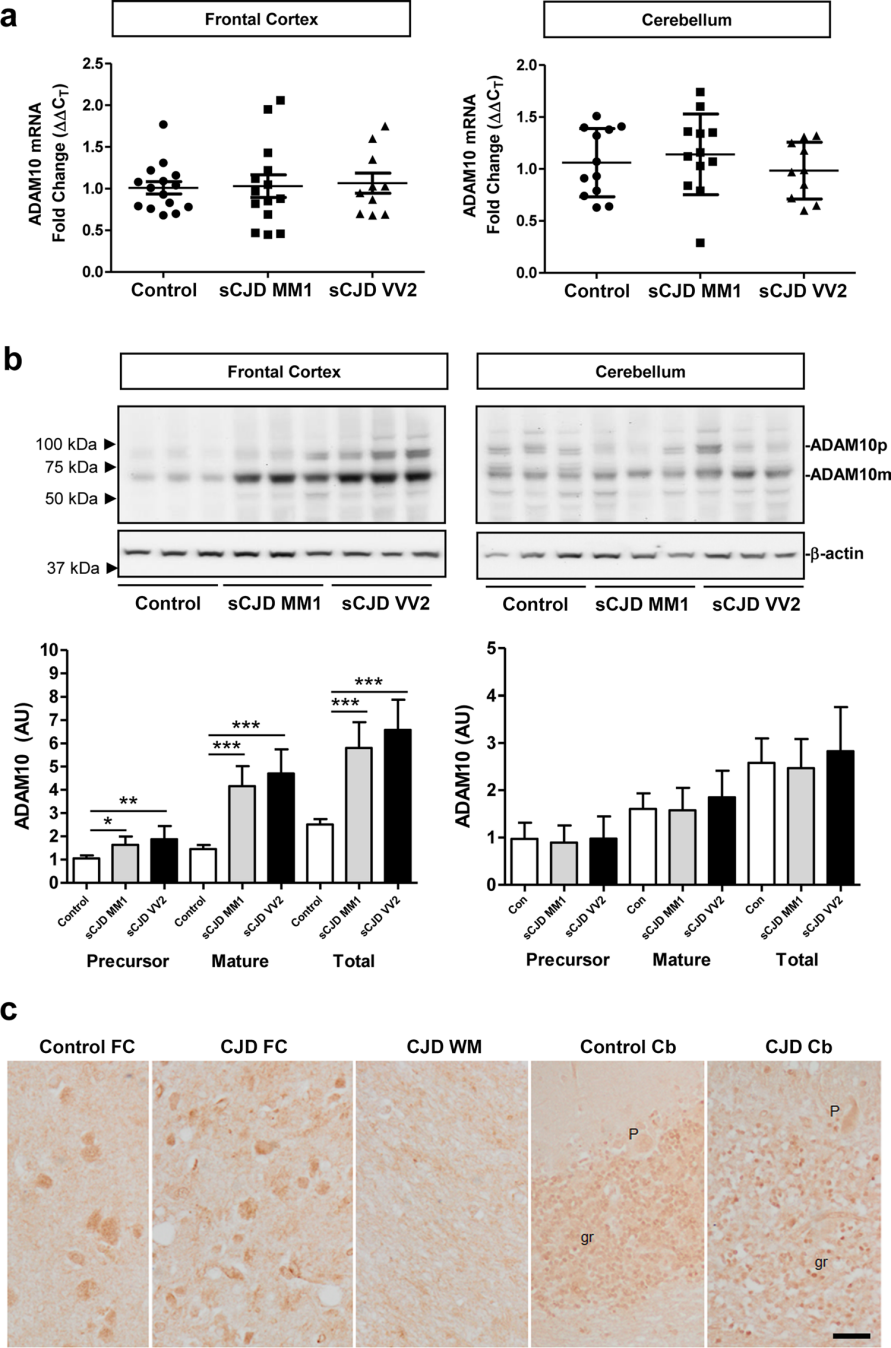
ADAM10 expression in sCJD brain tissue. **a** RT-qPCR analysis of *ADAM10* in the frontal cortex (FC) and cerebellum (CB) of control, sCJD MM1, and sCJD VV2 cases. *GAPDH* was used for normalization. FC (controls *n* = 15, sCJD MM1; *n* = 11, sCJD VV2 *n* = 10), CB (controls *n* = 12, sCJD MM1; *n* = 12, sCJD VV2 *n* = 10). **b** Representative western blot analysis of ADAM10 in the FC and CB of control, sCJD MM1, and sCJD VV2 cases. ADAM10 precursor (ADAM10p) and mature (ADAM10m) forms are indicated. β-actin was used for normalization. Graphical representation of western blot data acquired from the analysis of FC (controls *n* = 12, sCJD MM1; *n* = 12, sCJD VV2; *n* = 12) and CB (controls; *n* = 9, sCJD MM1; *n* = 10, sCJD VV2; *n* = 10); *p < 0.05, **p < 0.01, ***p < 0.001. **c** ADAM10 immunohistochemistry in the frontal cortex (FC), cerebellum (Cb), and subcortical white matter (WM) in control and sCJD MM1. ADAM10 immunoreactivity is observed in neurons in the FC and granule cells (gr) of cerebellum, but Purkinje cells (P) are very weakly stained or not at all. No positive cells are distinguished in the white matter; scale bar = 20 µm

Next, we explored whether ADAM10, the main TREM2 sheddase responsible for its regulated intramembrane proteolysis [60], was also altered in sCJD patients. With RT-qPCR analysis, no alterations in *ADAM10* mRNA levels were observed in sCJD, either in the FC (controls, *n* = 15; sCJD MM1, *n* = 14 and sCJD VV2, *n* = 10) or in the CB (controls, *n* = 12; sCJD MM1, *n* = 12 and sCJD VV2, *n* = 10) (Fig. 2a). *TREM2* levels were normalized to *GAPDH*, and similar results were obtained for *GUSB* normalization (data not shown). In contrast, ADAM10 precursor and mature forms were increased in western blot in the FC of sCJD MM1 (*p* < 0.05 precursor, *p* < 0.001 mature) and sCJD VV2 (*p* < 0.01 precursor, *p* < 0.001 mature) compared to controls (Fig. 2b). In the CB, ADAM10 levels were not significantly different for sCJD compared to controls (*p* > 0.05 in all comparisons) (Fig. 2b). In both brain regions, 10 controls, 8 sCJD MM1, and 8 sCJD VV2 cases were used. The ADAM10 antibody showed suboptimal results for immunohistochemistry; ADAM10 immunostaining was weak and blurred. ADAM10 immunoreactivity was moderate in neurons of the FC, weaker in the granular cells of the cerebellum and negative in Purkinje cells, in control and sCJD MM1 cases (Fig. 2c). sCJD VV2 was not analysed. Incubation with only the secondary antibody showed no immunoreactions (data not shown).

### Regional and temporal TREM2 expression in the brain of sCJD mouse models

**Fig. 3:**
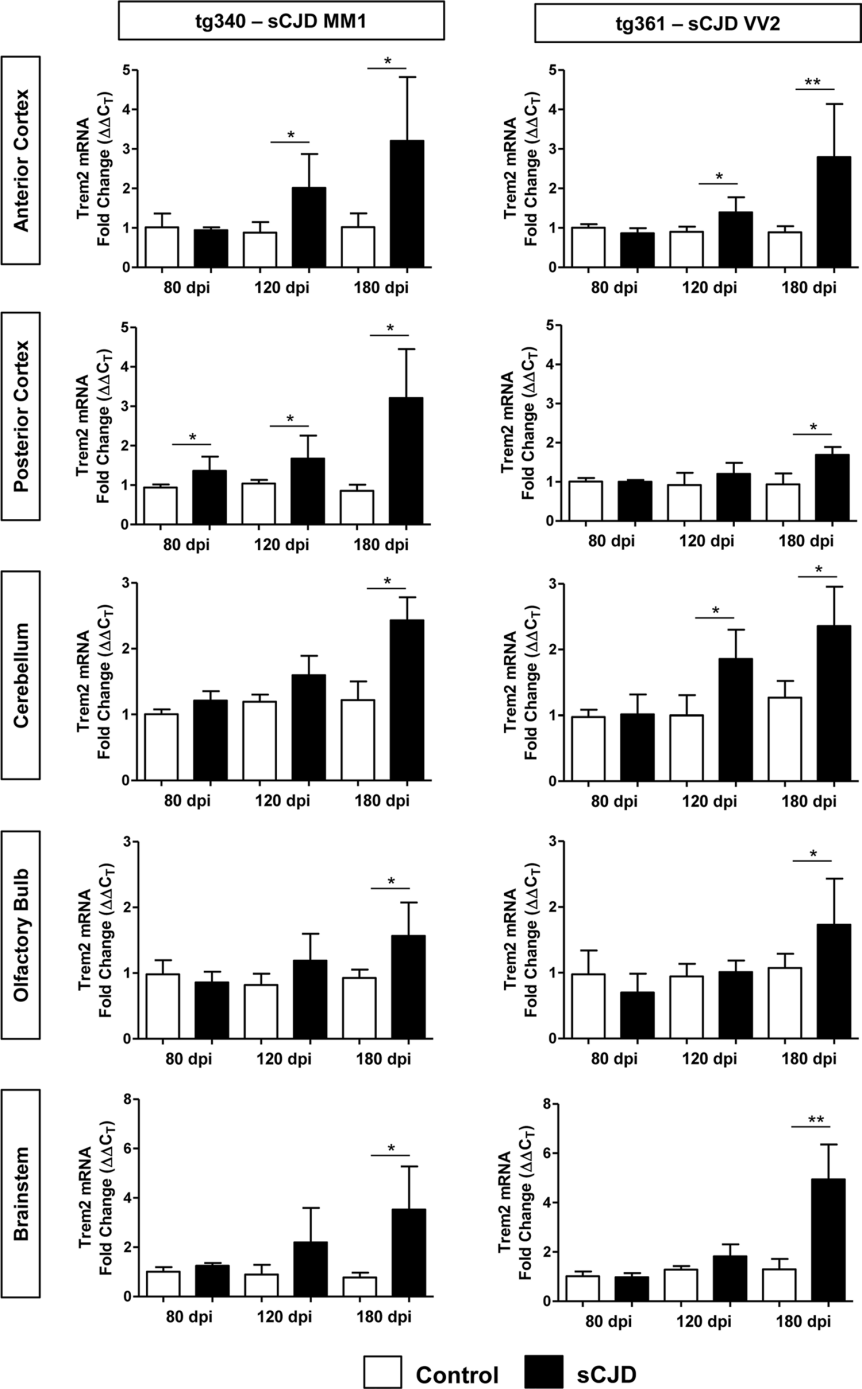
mRNA Trem2 expression in the brain of sCJD MM1 and VV2 mouse models. RT-qPCR analysis *Trem2* expression in mouse models of sCJD MM1 (tg340-CJD MM1) (left panels) and sCJD VV2 (tg361-CJD VV2) (right panels) subtypes. Trem2 expression was analysed in five brain regions: anterior and posterior cortex, cerebellum, olfactory bulb, and brainstem, at three time points: pre-clinical (80 days post inoculation (dpi)), early-clinical (120 dpi), and clinical (180 dpi). CJD-inoculated animals (black columns) were compared to their corresponding control-inoculated animals (white columns). Four to eight animals per time point and condition were used. Normalization was performed using the housekeeping gene *Hprt*; *p < 0.05, **p < 0.01

To investigate the regional and temporal alterations of TREM2 during prion pathogenesis in brain tissue, we took advantage of two sCJD mouse models recapitulating the molecular and pathological alterations of sCJD MM1 (tg340-sCJD MM1) and sCJD VV2 (tg360-sCJD VV2) subtypes [9, 10, 34]. *Trem2* mRNA expression was investigated in the anterior and posterior cortex, cerebellum, olfactory bulb, and brainstem at pre-clinical (80 dpi), early clinical (120 dpi), and clinical (180 dpi) disease stages (*n* = 4–8) (Fig. 3). At the clinical stage, *Trem2* mRNA was increased in all brain regions in both CJD models. Increased *Trem2* expression was also observed at early-clinical stages in the anterior cortex of both models. Differences between sCJD models were detected in the posterior cortex, where *Trem2* was specifically increased at preclinical and early-clinical stages in the tg340 sCJD model and in the cerebellum, where *Trem2* mRNA expression was specifically increased at early-clinical stages in the tg361 sCJD model (Fig. 3).

### sTREM2 in the CSF of prion diseases

The presence of increased TREM2 expression and ADAM10 expression and activation in the brain of sCJD raised the possibility that sTREM2 could be differentially altered in biological fluids of sCJD cases. CSF TREM2 concentrations were assessed in HC, ND, prion diseases from sporadic, genetic and acquired aetiology, AD, and MS (Table 1). sTREM2 was associated neither with age at onset nor with sex (Table 1), both when all cases were analysed together and when stratified between non-prion and prion diseases.

**Fig. 4:**
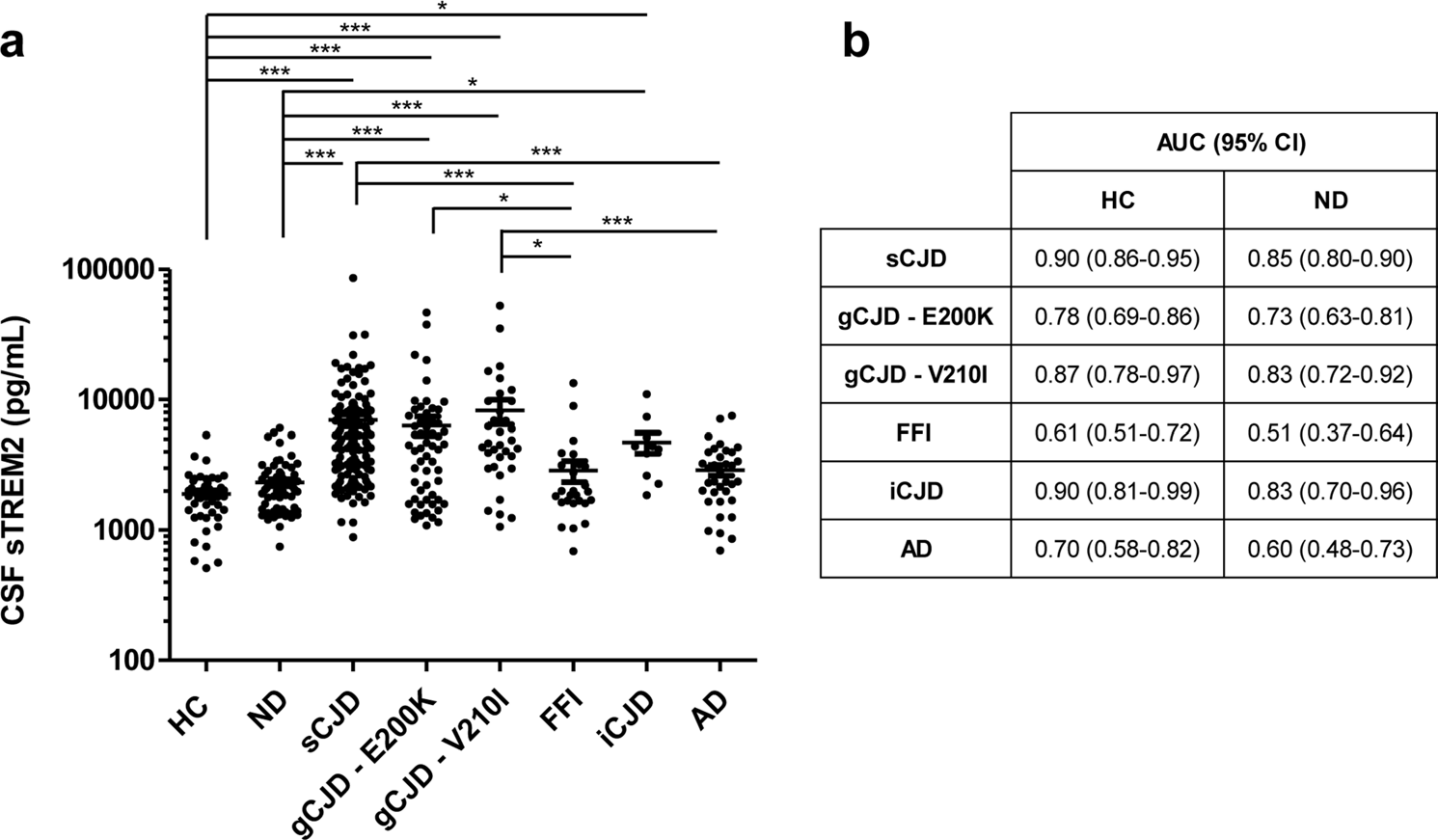
CSF sTREM2 in the diagnosis of prion diseases. **a** CSF sTREM2 concentrations in HC (*n* = 48), ND (*n* = 64), sCJD (*n* = 139), gCJD-E220K (*n* = 57), gCJD-V210 (*n* = 34), FFI (*n* = 26), iCJD (*n* = 10), and AD (*n* = 35). **b** Area under the curve (AUC) derived from receiver operating characteristic (ROC) curves with 95% confidence interval (95% CI) for CSF sTREM2 in the discrimination of prion diseases and AD from HC and ND; *p < 0.05, ***p < 0.001

Compared to HC and ND, sTREM2 concentrations were significantly higher in all types of prion diseases, with the exception of FFI cases (Fig. 4a). sTREM2 mean concentrations were higher in ND (2413 ± 1262 pg/mL) than in HC (1901 ± 878 pg/mL). Accordingly, the AUC derived from the sCJD vs. HC comparison (0.90) was higher than that for the sCJD vs. ND comparison (0.85) (Fig. 4b). An optimal cut-off of 2650 pg/mL sTREM2 discriminated HC from sCJD cases with 79% sensitivity and 94% specificity, while a cut-off of 2850 pg/mL sTREM2 discriminated ND from sCJD cases with 77% sensitivity and 80% specificity. AUCs for the discrimination of gCJD E200K, gCJD V210I, and iCJD from HC and ND showed a good diagnostic accuracy ranging from 0.78 to 0.90 (compared to HC) and from 0.73 to 0.85 (compared to ND). In contrast, sTREM2 showed no diagnostic value in distinguishing HC and ND from FFI cases (AUC 0.62 and 0.51, respectively) (Fig. 4b). sTREM2 was also quantified in AD cases. Mean sTREM2 concentrations in AD were slightly higher than in HC and ND; however, differences did not reach statistical significance (Table 1 and Fig. 4a). In contrast, sTREM2 levels were significantly higher in sCJD and gCJD-V210I compared to AD (Fig. 4a). sTREM2 levels were also assessed in two series of MS cases using different populations and different methods. sTREM2 levels in the CSF were not altered in MS when compared with controls (data not shown).

**Fig. 5:**
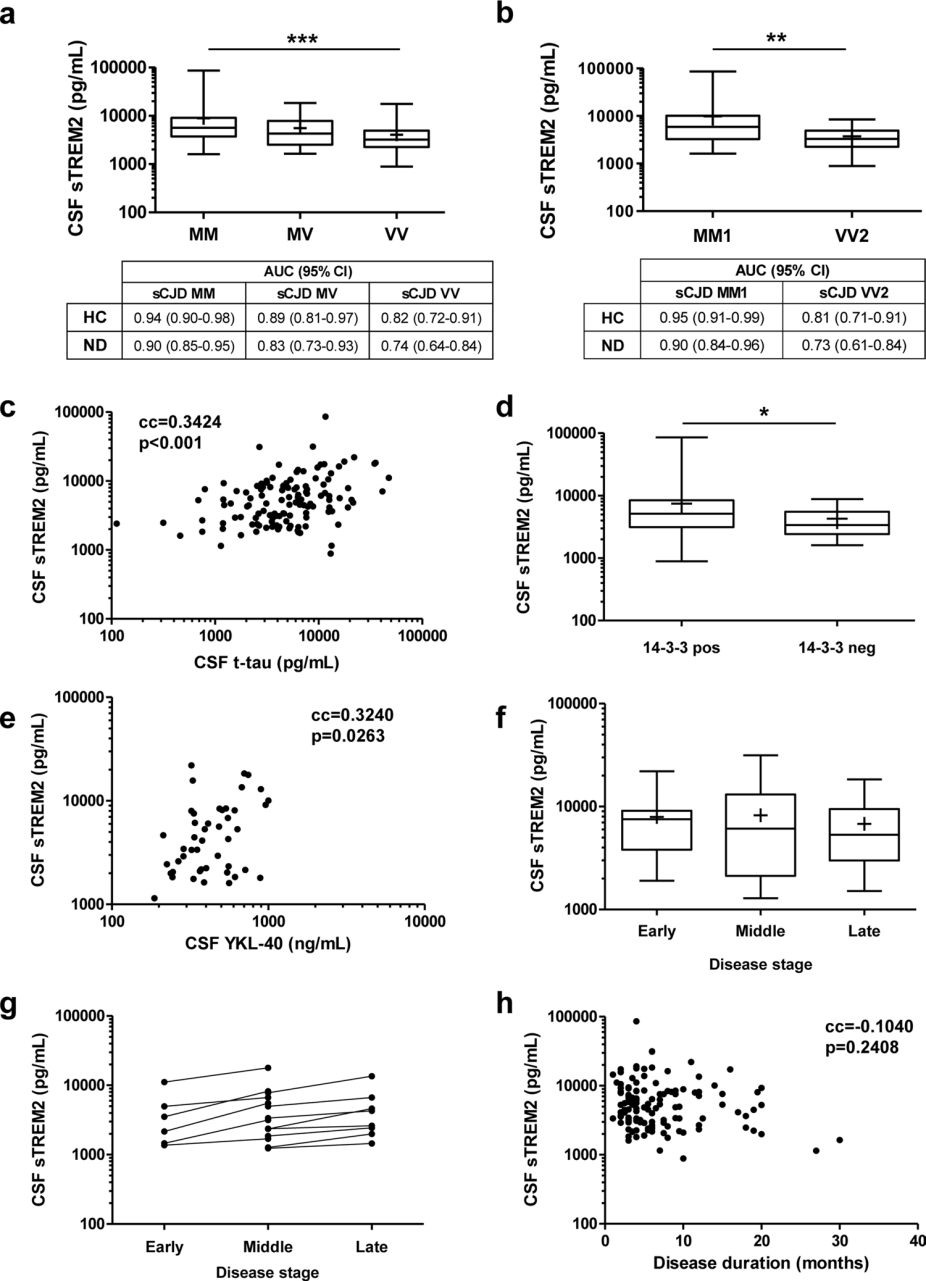
CSF sTREM2 in sCJD: association with codon129 genotype, sCJD subtype, CSF biomarkers, and clinical data. **a** CSF sTREM2 concentrations stratified by prion protein gene (*PRNP*) codon 129 polymorphism (M = methionine, V = valine). sCJD MM; *n* = 32, sCJD MV; *n* = 19 and sCJD VV; *n* = 17. Mean ( +), median (horizontal bar inside the box) and interquartile ranges are represented. Area under the curve (AUC) values with 95% confidence intervals (95% CI) indicated for each comparison. **b** CSF sTREM2 concentrations stratified by sCJD subtype MM1 (*n* = 20) and VV2 (*n* = 9). Mean ( +), median (horizontal bar inside the box), and interquartile ranges are represented. Area under the curve (AUC) values with 95% confidence intervals (95% CI) indicated for each comparison. **c** Correlation analysis between CSF TREM2 and CSF t-tau (*n* = 126). **d** Association analysis of CSF TREM2 concentrations with CSF 14-3-3 positivity. 14-3-3 positive cases; *n* = 118 and 14-3-3 negative cases; *n* = 21. Mean ( +), median (horizontal bar inside the box), and interquartile ranges are represented. **e** Correlation analysis between CSF sTREM2 and CSF YKL-40 (*n* = 47). **f** CSF sTREM2 concentrations stratified by disease stage at the time of lumbar puncture (LP). Samples were grouped into three categories according to whether they underwent LP in the first (< 0.33), second (0.33–0.66) or third (> 0.66) stage of the disease. Early disease stage, *n* = 15; middle disease stage, *n* = 21; late disease stage, *n* = 60. Mean ( +), median (horizontal bar inside the box), and interquartile ranges are represented. **g** Longitudinal analysis of CSF TREM2 concentrations in cases with available LP at different disease stages (*n* = 14). **h** Correlation analysis between sTREM2 concentrations and disease duration (months) (*n* = 126). cc: correlation coefficient; *p < 0.05, **p < 0.01, ***p < 0.001

Next, we explored the influence of codon 129 polymorphism in *PRNP* on sTREM2 levels. The highest sTREM2 concentrations were detected in sCJD cases harbouring methionine/methionine (MM; 7884 ± 6652 pg/mL, *n* = 32), followed by methionine/valine (MV; 5257 ± 4061 pg/mL, *n* = 19) and valine/valine (VV; 4183 ± 3803 pg/mL, *n* = 17). Statistical differences were detected between MM and VV cases (*p* = 0.03) (Fig. 5a). pROC analysis revealed a superior CSF sTREM2 diagnostic accuracy for sCJD MM compared to sCJD VV cases, both when compared to HC (AUC = 0.94 vs 0.82, *p* = 0.0177) and to ND (AUC = 0.90 vs. 0.74, *p* = 0.0076) (Fig. 5a).

We further evaluated CSF sTREM2 in the two most prevalent sCJD subtypes (MM1 and VV2) in those cases with prion type available from autopsy analysis. sCJD MM1 (8196 ± 7782 pg/mL, *n* = 20) presented higher sTREM2 concentrations than sCJD VV2 cases (3224 ± 2200 pg/mL, *n* = 9) (Fig. 5b). In line with these observations, AUC values were significantly different between the two subtypes when compared to HC (AUC = 0.95 vs 0.81, *p* = 0.0151) and ND (AUC = 0.90 vs. 0.73, *p* = 0.0070) (Fig. 5b), indicating a significantly higher diagnostic value of sTREM2 in the discrimination of sCJD MM1 cases. Regarding genetic prion diseases, gCJD-E200K and FFI cases were stratified according to their *PRNP* codon 129 genotype, which influences their clinicopathological features [17, 47]. sTREM2 concentrations were not different between gCJD-E200K-MM (*n* = 33) and gCJD-E200K-MV (*n* = 22) cases (*p* = 0.59) nor between FFI-MM (*n* = 16) and FFI-MV (*n* = 10) cases (*p* = 0.61) (data not shown).

Cerebrospinal fluid tests sTREM2 in sCJD displayed a positive correlation with *t*-tau (*n* = 126, cc = 0.3424, *p* < 0.001) (Fig. 5c), and was associated with 14-3-3 positivity, since sCJD cases presenting a positive 14-3-3 test (*n* = 118) showed higher sTREM2 concentrations than those with a negative (or inconclusive) 14-3-3 test (*n* = 21) (*p* = 0.03) (Fig. 5d). Additionally, sTREM2 correlated with YKL-40 concentrations (*n* = 47, cc = 0.3240, *p* = 0.0263) (Fig. 5e).

To investigate a potential influence of the disease stage at which the LP was performed and sTREM2 concentrations in sCJD patients, samples were stratified in early (7914 ± 5924 pg/mL, *n* = 9), middle (8229 ± 7854 pg/mL, *n* = 20), and late (6794 ± 4950 pg/mL, *n* = 35) stages, but no significant differences were detected between groups (Fig. 5f). In 14 individuals, serial LPs at different disease stages were available allowing investigation of whether sTREM2 values increased during disease progression (Fig. 5g). Using a multilevel linear model, we observed a significant increase of 1962 pg/mL (95% CI 811–3113) per disease stage (*p* = 0.0036). CSF sTREM2 concentrations were not significantly associated with total disease duration (cc = − 0.1040, *p* = 0.2408) (Fig. 5h), ruling out a potential role of CSF sTREM2 as sCJD prognostic marker.

### sTREM2 in the plasma of sCJD

**Fig. 6:**
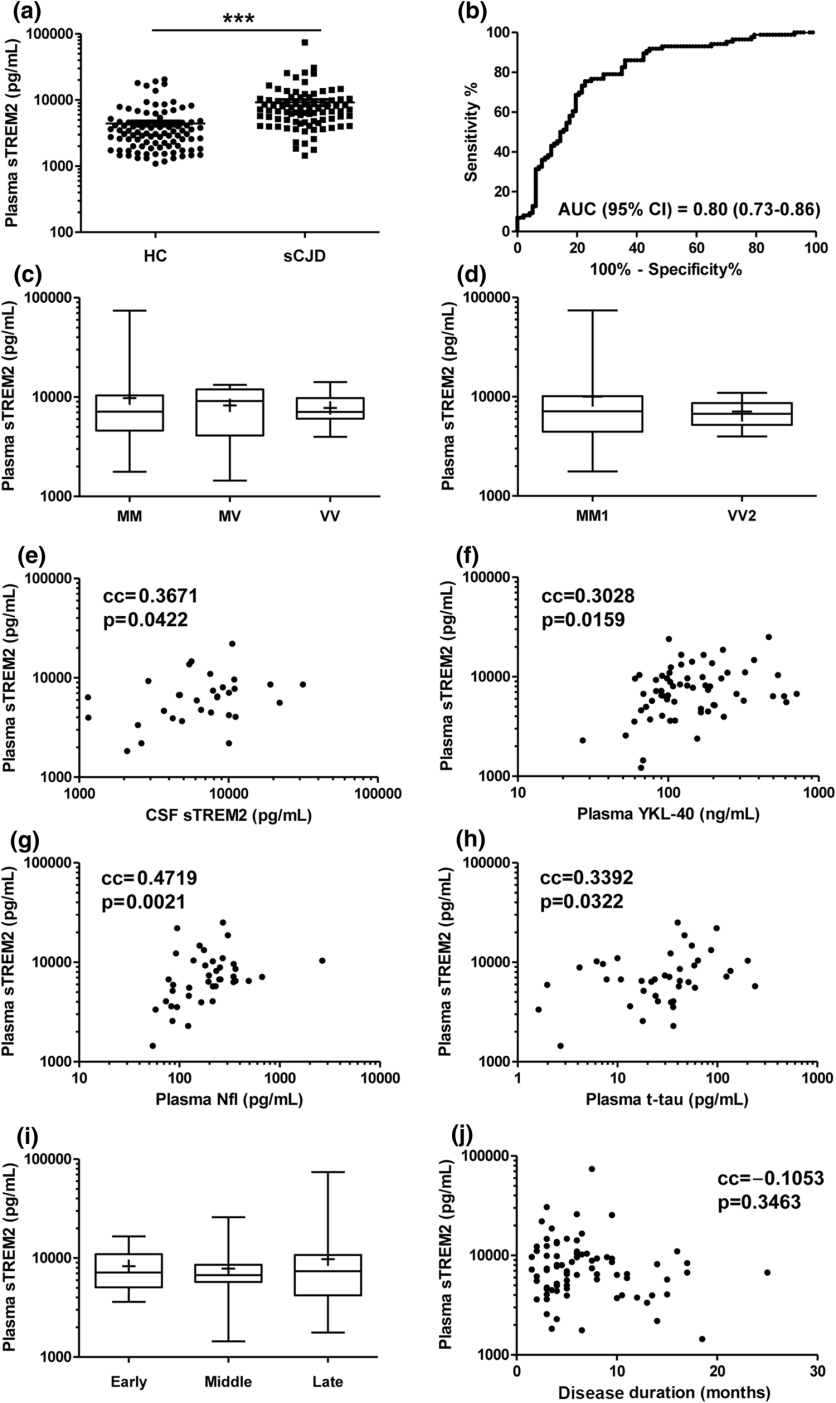
Plasma sTREM2 in sCJD: association with codon129 genotype, sCJD subtype, plasma biomarkers, and clinical data. **a** Plasma sTREM2 concentrations in HC (*n* = 97) and sCJD (*n* = 86) cases. **b** Area under the curve (AUC) derived from receiver operating characteristic (ROC) curves with 95% confidence interval (95% CI) for plasma sTREM2 in the discrimination of sCJD from HC. **c** Plasma sTREM2 concentrations stratified by prion protein gene (*PRNP*) codon 129 polymorphism (M = methionine, V = valine). sCJD MM; *n* = 59, sCJD MV; *n* = 8 and sCJD VV; n = 14. Mean ( +), median (horizontal bar inside the box), and interquartile ranges are represented. **d** Plasma sTREM2 concentrations stratified by sCJD subtype MM1 (*n* = 45) and VV2 (*n* = 12). Mean ( +), median (horizontal bar inside the box) and interquartile ranges are represented. **e** Correlation analysis between plasma and CSF sTREM2 (*n* = 31). Correlation analysis between plasma sTREM2 and plasma YKL-40 **f** (*n* = 63), plasma Nfl (*n* = 40) **g** and plasma *t*-tau (*n* = 40) **h. i** Plasma sTREM2 concentrations stratified by disease stage at the time of blood uptake. Samples were grouped into three categories according to whether they underwent blood uptake in the first (< 0.33), second (0.33–0.66), or third (> 0.66) stage of the disease. Early disease stage, *n* = 14; middle disease stage, *n* = 15; late disease stage, *n* = 53. Mean ( +), median (horizontal bar inside the box), and interquartile ranges are represented. **j** Correlation analysis between plasma sTREM2 concentrations and disease duration (months) (*n* = 82). cc: correlation coefficient; ***p < 0.001

Soluble TREM2 concentrations in plasma have been reported to be in the same range as detected in the CSF [52]. Thus, we explored whether sTREM2 in plasma could be altered in sCJD compared to HC. Plasma sTREM2 in HC and sCJD was associated neither with age at onset nor with sex (Table 2). In sCJD, sTREM2 (8981 ± 8932 pg/mL) was significantly increased compared to HC (4409 ± 3961) (*p* < 0.001) (Table 2, Fig. 6a), with an AUC value of 0.80 in the discrimination of both diagnostic groups (Fig. 6b).

sTREM2 concentrations in sCJD were associated neither with *PRNP* codon 129 genotype: MM (9755 ± 10,492 pg/mL, *n* = 59), MV (8238 ± 4220 pg/mL, *n* = 8) and VV (7787 ± 2773 pg/mL, *n* = 14) (*p* > 0.05 in all comparisons) (Fig. 6c) nor with sCJD subtype: MM1 (10,040 ± 11,570, *n* = 45) and VV2 cases (7106 ± 2147, *n* = 12) (*p* = 0.8654) (Fig. 6d). In a subset of sCJD cases where plasma-CSF paired data were available (*n* = 31), sTREM2 showed a significant correlation between the fluids (cc = 0.3671, *p* = 0.0422) (Fig. 6e). Plasma sTREM2 showed a modest positive correlation with plasma YKL-40 (*n* = 63, cc = 0.2958, *p* = 0.0217) (Fig. 6f) Nfl (*n* = 40, cc: 0.4719, *p* = 0.0021) (Fig. 6g) and *t*-tau (*n* = 40, cc = 0.3392, *p* = 0.0322) (Fig. 6h) concentrations.

No significant differences in plasma sTREM2 were detected between the different disease stages at which blood was obtained: early (8258 ± 3842 pg/mL, *n* = 14), middle (7852 ± 5371 pg/mL, *n* = 15), and late (9768 ± 10,699 pg/mL, *n* = 53) stages (*p* > 0.05 in all comparisons) (Fig. 6i).

Similar to CSF sTREM2, plasma sTREM2 was not associated with sCJD disease duration (cc = − 0.1053, *p* = 0.3463) (Fig. 6j).

## Discussion

In the present study, we report TREM2 expression and levels in the brain and biological fluids of prion diseases, with special focus on sCJD, uncovering its potential role as a diagnostic marker. In sCJD, we observed a significant increase in *TREM2* mRNA compared with controls and FTLD-TDP43. TREM2 protein is increased in the FC and CB of sCJD post-mortem-human brain tissue at the protein level (both in its immature and mature forms), as revealed with western blotting. Immunohistochemistry using the anti-TREM2 antibody from RD Systems (AF1828) shows TREM2 expression in blood vessels and microglia. Neurons are also stained using goat anti-TREM2 Abcam antibody in our cases. TREM2 immunoreactivity has been reported in neurons, astrocytes, and oligodendrocytes using goat anti-TREM2 Abcam antibody in another study [11]. Since there is no evidence of neuronal TREM2 mRNA production in neurons, and the Abcam antibody is discontinued, we assume that additional studies are needed to postulate TREM2 as a neuronal marker.

The positive correlation between *TREM2* and the macrophage marker CD68 at the mRNA level supports immunohistochemical findings and suggests that microglia account for the increased TREM2 expression in sCJD brain. This observation, in conjunction with increased ADAM10 expression and activation, points to a potential increase of sTREM2 in biological fluids of prion diseased patients.

In agreement with this hypothesis, sTREM2 displays good accuracy in discriminating sCJD from ND (AUC = 0.85) and HC (AUC = 0.90) in CSF, and from HC (AUC = 0.80) in plasma. This despite diagnostic accuracies being lower than those reported for CSF biomarkers currently used in clinical settings (14-3-3, *t*-tau (*p*-tau/*t*-tau) and RT-QuIC) [13, 36, 44, 62]. The CSF sTREM2 increase in gCJD and iCJD is in line with the similar clinicopathological features of both conditions with sCJD, and consequently, with their fluid biomarkers profiles [32, 38]. In contrast, CSF sTREM2 is not altered in FFI, in agreement with the low sensitivity of prion disease biomarkers in this condition [40], most likely reflecting the restricted pathological alterations in terms of neuroinflammation and longer disease duration compared with CJD cases [8].

Previous studies have shown slightly or moderately increased sTREM2 levels in the CSF in patients with AD [22, 24, 55, 67, 68]. Moreover, individuals with MS and other inflammatory CNS diseases show increased CSF sTREM levels [1, 48, 78]. However, we have not found significant differences in the CSF levels of sTREM2 in AD and MS cases in our series. We can interpret these discrepancies due to lower discrimination capacity of our methods when compared with those used in other labs. If this hypothesis is true, the present findings reinforce the magnitude of sTREM alterations in prion diseases when compared with AD and MS.

Interestingly, and in contrast to TREM2, sCJD neuroaxonal damage markers with exclusive neuronal expression such as *t*-tau and 14-3-3 display reduced protein levels in sCJD brain tissue [39]. CSF sTREM2 correlates with the neuronal damage markers *t*-tau and 14-3-3 and with the astrocytic marker YKL-40. Similarly, plasma sTREM2 positively correlates with YKL-40, *t*-tau, and Nfl. The moderate but significantly positive association between CSF and plasma sTREM2 in sCJD cases, similar to what is observed in AD [4], suggests that plasma sTREM2 may reflect the CNS neuroinflammatory profile in these conditions. However, the reported expression of TREM2 in non-CNS tissues [14, 74], especially in pathological conditions, does not rule out a potential contribution of these tissues to plasma sTREM2 concentrations and could explain the differing influence of *PRNP* codon 129 and sCJD subtype between CSF and plasma.

The influence of *PRNP* codon 129 on CSF sTREM2 (higher concentrations in MM compared to VV cases) is different to that reported for neuronal damage markers such as CSF t-tau and 14-3-3, which display significantly lower values in CJD MV cases compared to homozygotes [19, 27], and is explained by the less severe phenotype and longer disease duration of CJD MV cases.

Importantly, the increased CSF TREM2 concentrations in sCJD MM compared to sCJD VV cases led to significantly better AUC values in MM cases, reaching high discriminatory power compared to controls (HC, AUC = 0.94 and ND, AUC = 0.90). Thus, data on *PRNP* polymorphism (routine test in prion diagnostic centres) may improve the accuracy of CSF sTREM2 in terms of disease diagnosis and should be taken into consideration in patient stratification when used as a potential surrogate endpoint in disease-modifying therapy.

The reason for increased CSF sTREM2 in sCJD MM1 compared to sCJD VV2 remains unknown, but it could be explained by the higher microglial profile in the cortical regions of sCJD MM1 compared with VV2 cases [34]. This, together with the observation that increased TREM2 expression is more significant in FC than in CB, suggests that sTREM2 derived from cortical regions is the main contributor to its increased concentrations in biological fluids.

Another interesting finding in our study is the observation that sCJD mouse models have increased TREM2 levels, occurring in some brain regions at pre-clinical and early-clinical stages in a regional and disease subtype-specific manner. This is in line with the observation that CSF sTREM2 concentrations are sCJD-subtype-specific and independent of the disease stage at which sampling is performed. It is therefore tempting to speculate that sTREM2 alterations in biological fluids may occur at early disease stages. Unfortunately, the absence of a prodromal stage of sCJD precludes the study of pre-clinical cases and leaves this possibility open for the analysis of sTREM2 in asymptomatic gCJD cases associated with the E200K and V210I mutations.

In contrast with longitudinal data from neuroaxonal/synaptic damage markers such as t-tau, 14-3-3, Nfl, and α-syn, which present marginal or absent alterations of their CSF concentrations along disease progression [33, 59, 77], the progressive neuroinflammatory profile in sCJD brain may explain increased CSF sTREM2 concentrations in follow-up LPs, suggesting a potential role for sTREM2 as a candidate marker of microglial activation. The observation that CSF and plasma sTREM2 are not associated with disease duration in sCJD precludes a prognostic value for sTREM2.

In conclusion, we have found expression of TREM2 in a subpopulation of microglial cells in human sCJD brains, with increased mRNA and protein levels in brain compared to controls. Further, we have shown that CSF and plasma sTREM2 have diagnostic accuracy in the discrimination of sCJD. We may speculate that sTREM2 quantification in CSF may be useful for complementary diagnostic purposes. However, the assessment of TREM2 levels in the CSF in other neurological diseases, that may represent clinical diagnosis difficulties with CJD, is a pending condition. Unfortunately, adequate numbers of samples from cases with paraneoplastic encephalitis, other rare encephalitis, and rapid forms of non-prionic dementia were not available in the present study. Finally, our data highlight the importance of molecular neuropathology assessment in the context of biomarker research toward understanding of the aetiology of the alterations detected in biological fluids, which in turn may be relevant in the interpretation of the translational value of biomarker profiles.

## Supplementary Information

Below is the link to the electronic supplementary material.

Supplementary file1 (DOCX 57 KB)

## Declarations

### Human sample acquisition

Brain tissue was obtained from the Institute of Neuropathology HUB-ICO-IDIBELL Biobank and the Hospital Clinic de Barcelona-IDIBAPS Neurological Tissue Bank following the Spanish legislation guidelines on this matter (Real Decreto de Biobancos 1716/2011) and approval of the local ethics committees. CSF samples were collected at the following centres: (1) Clinical Dementia Centre and the National Reference Centre for CJD Surveillance at the University Medical Centre of Göttingen (Germany); (2) Alzheimer's Disease and Other Cognitive Disorders Unit, Hospital Clínic de Barcelona, Barcelona (Spain); (3) Multiple Sclerosis Unit Neurology Service, Bellvitge University Hospital, Hospitalet de Llobregat, Spain; (4) National Centre of Microbiology-Instituto de Salud Carlos III, Madrid, Spain; and (5) Istituto Superiore di Sanità, Rome, Italy. Blood was collected at the Department of Transfusion Medicine (healthy controls) and at the Department of Neurology (sCJD cases) of the University Medical Centre Göttingen (Germany) under standardized pre-analytical conditions.

### Animal experimentation

We conducted animal experiments in accordance with the Code for Methods and Welfare Considerations in Behavioural Research with Animals (Directive 2010/63/EU) and made every effort to minimize suffering. Experiments developed in the Centro de Investigación en Sanidad Animal–Instituto Nacional de Investigación y Tecnología Agraria y Alimentaria (Madrid, Spain) were evaluated by the Committee on the Ethics of Animal Experiments of the Instituto Nacional de Investigación y Tecnología Agraria y Alimentaria and approved by the General Directorate of the Madrid Community Government (permit no. PROEX 094/18).
