# Supplementary material for: TREM2 expression in the brain and biological fluids in prion diseases
Source: Acta Neuropathol. 2021 Apr 21;141(6):841–59. doi: 10.1007/s00401-021-02296-1 (PMC8113222; doi:10.1007/s00401-021-02296-1)
Supplement: Supplementary file 1 — Supplementary file1 (DOCX 57 KB) [file 401_2021_2296_MOESM1_ESM.docx]

**Supplementary table 1:** Summary of cases used for qPCR studies. Cases demographic (age and sex (F=female, M=male)), post-mortem delay time (PMT) and RIN data per gene analysed of CJD FC and CB group, and sFTLD group.

**Supplementary table 2:** Summary of cases used in each western blot study. Cases used for each protein detection of controls and sCJD (MM1 and VV2) FC and CB.

**Supplementary table 3:** De-waxed paraffin sections used in IHC/IF. Demographic data (age and sex (f=female, m=male)) and post-mortem delay time (PMT). MS and Vascular dementia Pending

**Supplementary table 4:** Cerebrospinal fluid samples demographic data (age and sex (f=female, m=male))
